# Connecting systemic inflammation to prognosis in neuroendocrine neoplasms: a biomarker-based approach

**DOI:** 10.3389/fmed.2026.1728726

**Published:** 2026-02-16

**Authors:** Andreea Iliesiu, Ancuța-Augustina Gheorghişan-Gǎlǎțeanu, Dana Antonia Țǎpoi, Ioana Maria Lambrescu, Mariana Costache, Luiza Elena Tomescu, Diana Derewicz, Vanda Roxana Nimigean

**Affiliations:** 1 Department of Pathology, Carol Davila University of Medicine and Pharmacy, Bucharest, Romania; 2 Department of Pathology, University Emergency Hospital, Bucharest, Romania; 3 Department of Cellular and Molecular Biology and Histology, Carol Davila University of Medicine and Pharmacy (Ret.), Bucharest, Romania; 4 Parhon National Institute of Endocrinology (Ret.), Bucharest, Romania; 5 Department of Pathology, Synevo, Bucharest, Romania; 6 Department of Cellular and Molecular Biology and Histology, Carol Davila University of Medicine and Pharmacy, Bucharest, Romania; 7 Victor Babes National Institute of Pathology, Bucharest, Romania; 8 Department of Pediatrics, Carol Davila University of Medicine and Pharmacy, Bucharest, Romania; 9 Department of Pediatric Hematology and Oncology, Marie Sklodowska Curie Clinical Emergency Hospital, Bucharest, Romania; 10 Department of Oral Rehabilitation, Faculty of Dentistry, Carol Davila University of Medicine and Pharmacy in Bucharest, Bucharest, Romania

**Keywords:** inflammation, neuroendocrine neoplasms, neutrophil-to-lymphocyte ratio, platelet-to-lymphocyte ratio, prognostic biomarkers, systemic immune-inflammation index

## Abstract

**Background:**

Neuroendocrine neoplasms (NENs) comprise a diverse group of tumors with distinct clinical courses and prognoses. However, there remains a need to identify affordable biomarkers that can augment traditional prognostic indicators.

**Methods:**

To address this clinical need, we performed a retrospective analysis of 60 patients with NENs treated at the University Emergency Hospital of Bucharest (2016–2023). The baseline neutrophil-to-lymphocyte ratio (NLR), platelet-to-lymphocyte ratio (PLR), and systemic immune-inflammation index (SII) were derived from routine blood counts and were associated with demographic, histopathological, and mortality during follow-up. Logistic regression models assessed prognostic significance.

**Results:**

Of the 60 patients, 38.3% died during follow-up. Univariate analysis identified low lymphocyte counts, elevated platelet counts, PLR, NLR and SII as significant predictors of mortality. Building upon these findings, multivariate models revealed that advanced age, poorly differentiated NEC histology, and elevated SII were significantly associated with increased mortality.

**Conclusion:**

In summary, this study identifies SII, NLR and PLR as accessible, reliable, and cost-effective biomarkers with prognostic value in NENs. Thus, integrating these indices with established clinicopathological features may improve risk stratification and inform personalized management. Nonetheless, validation in larger, prospective cohorts is necessary to substantiate these findings.

## 1 Introduction

Neuroendocrine neoplasms (NENs) are malignant tumors that originate from neuroendocrine cells. The phenotypic spectrum extends from poorly differentiated neuroendocrine carcinomas (NECs) to well-differentiated neuroendocrine tumors (NETs), which might explain the variation in clinical presentation and prognosis. Neuroendocrine tumors (NETs) are a rare category of malignancies characterized by slow development. These tumors exhibit endocrine activity and express neuroendocrine markers (1). Due to the widespread distribution of neuroendocrine cells, these tumors have been reported to occur in various parts of the body, with the digestive tract and lungs being the most common sites (2). Advancements in histology documentation and classification, imaging, and endoscopic procedures have driven a significant rise in age-adjusted incidence. Over the last several decades, rates have increased 3.7 times, from 2.35 to 8.61 per 100,000 persons (3).

Current guidelines employ different classifications for stratifying and following-up patients, considering tumor location, Ki-67, and specific tumor markers. Nevertheless, accurately assigning patients to a particular risk class is often challenging in clinical practice. Over the past 15 years, a new perspective on the molecular characterization of NENs has emerged, which may aid in identifying risk categories more effectively. However, not all facilities that treat these patients have access to molecular diagnostics due to their high cost. Biological mathematical modeling is a compelling field that integrates multiple variables to enhance the characterization and comprehension of specific diseases. This intricate analysis can also be applied to NENs, utilizing a variety of established biomarkers, as well as emerging indicators such as pretreatment inflammatory markers. For any physician who treats cancer patients, particularly those with NENs, the latter offer an affordable and convenient choice. Thus, the insight into the intricate biology of NENs and their related inflammatory markers can support the establishment of individualized therapeutic plans and lead to better clinical outcomes (4).

The novel concept of using inflammation-related counts to forecast tumor progression emerged from the interaction between inflammation and cancer. Recent research highlights the significance of evaluating pretreatment inflammation in cancer patients, as it is crucial to both the progression of the disease and the response to anticancer therapy (5). Thus, a simple blood count and the ratio between neutrophils (N), platelets (P), and lymphocytes (L) can provide significant insights into the prognosis of patients with oncological disease (6). Additionally, the systemic immune-inflammation index or SII (SII = N × P/L) has been recognized as being associated with the clinicopathological characteristics of tumors and the survival rates of a diverse array of malignancies (7).

In the present study, we investigated the prognostic significance of readily available inflammatory biomarkers, namely neutrophil-to-lymphocyte ratio (NLR), platelet-to-lymphocyte ratio (PLR), and systemic inflammation index (SII), in patients diagnosed with NENs. By correlating these systemic inflammatory indices with demographic, clinical, and histopathological parameters, we aimed to determine their potential role in predicting mortality during follow-up. Given their accessibility, low cost, and reproducibility, these markers may serve as complementary tools to traditional prognostic factors such as tumor differentiation, location, and invasion features, thereby supporting risk stratification and personalized management strategies in NENs.

## 2 Materials and methods

### 2.1 Study design and participants

This retrospective study included 60 patients with NENs recruited from the University Emergency Hospital of Bucharest between January 2016 and December 2023. Eligible patients were adults aged 18 or older with blood count information available prior to any therapy and with follow-up data. Patients with mixed tumors, other cancers aside from NENs, or signs of infection or inflammatory disease were excluded. Clinical data, follow-up details, and demographic information (age and gender) were collected from medical records.

In addition, we calculated several ratios: the neutrophil-to-lymphocyte ratio (NLR), which is calculated by dividing the absolute neutrophil count by the absolute lymphocyte count, and the lymphocyte-to-platelet ratio (PLR), which is calculated by dividing the absolute platelet count by the absolute lymphocyte count. Additionally, our investigation included the systemic inflammatory index (SII), which was calculated using the following formula: (Platelets × Neutrophils) ÷ Lymphocytes. A Hematology Analyzer was employed to determine the counts of neutrophils, lymphocytes, and platelets.

The study received the approval of the Ethics Committee of University Emergency Hospital (Approval No. 43323/16.06.2025). Informed consent was obtained from all patients included in the study, according to the Helsinki Declaration.

### 2.2 Statistical analysis

Categorical variables were expressed as frequencies and percentages and were compared using the χ^2^ test or Fisher’s exact test for low expected cell counts. Normality of continuous variables was assessed using the Shapiro–Wilk test and visual inspection of histograms. Continuous variables with normal distribution were presented as mean ± standard deviation and were compared using Student’s *t*-test. or median. Continuous variables with non-normal distribution are presented as median (interquartile range) and were compared using the Mann–Whitney U test.

Prognostic factors for mortality during follow-up were evaluated using logistic regression analysis. In univariate analysis, each clinical (age, gender, lymphovascular invasion [LVI], perineural invasion [PVI], tumor location, diagnosis) and laboratory parameter (neutrophils, lymphocytes, platelets, neutrophil-to-lymphocyte ratio (NLR), platelet-to-lymphocyte ratio (PLR), systemic immune–inflammation index (SII)) was tested separately. A Receiver Operating Characteristic (ROC) curve analysis to determine the optimal cut-off values for, neutrophils, lymphocytes, platelets, NLR, SII and PLR using the Youden Index. Patients were categorized into “High” vs. “Low” groups based on these cut-offs. For categorical variables, odds ratios were calculated using clinically relevant reference categories as follows: female sex, gastric tumor location, well-differentiated neuroendocrine tumor (NET), presence of lymphovascular invasion, and absence of perineural invasion. Multivariate logistic regression was performed for selected variables with a significant value in the univariate analysis to avoid overfitting and colinearity.

Results are presented as odds ratios (OR) with 95% confidence intervals (CI). An OR greater than 1 indicated an increased odd of death. Model fit was assessed using the Hosmer–Lemeshow goodness-of-fit test and Nagelkerke’s pseudo-*R*^2^. A two-tailed *p*-value <0.05 was considered statistically significant. Analyses were performed using GraphPad Prism version 10.6.0.

## 3 Results

The main demographic characteristics of the patients are presented in Table 1.

**Table 1:** Demographic characteristics of the study population.

Demographics	Age (Mean ± SD)	Gender		Living environment	
		Male	Female	Urban	Rural
Total	57.47 ± 15.63	40% (*n* = 24)	60% (*n* = 36)	68.33% (*n* = 41)	31.67% (*n* = 19)
Living	52.35 ± 15.08	43.24% (*n* = 16)	56.76% (*n* = 21)	75.68% (*n* = 41)	24.32% (*n* = 9)
Deceased	65.7 ± 12.98	34.78% (*n* = 8)	65.22% (*n* = 15)	56.52% (*n* = 13)	43.58% (*n* = 10)

**Figure 1:**
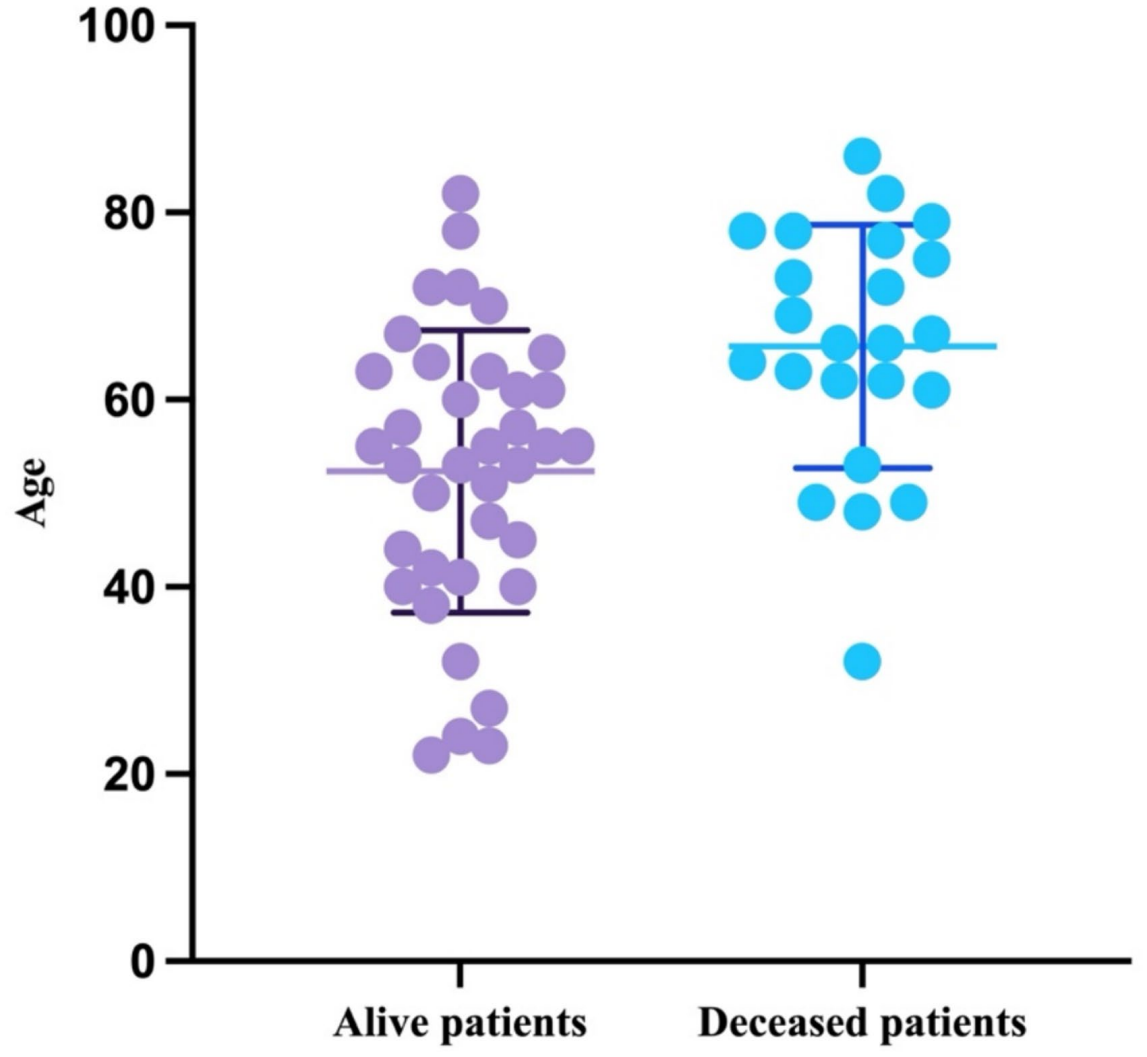
Scatter plot representing the mean with SD of ages of the patients based on the outcome.

The mean age of the patients included in this study was 57.47, with a standard deviation of 15.63; the youngest patient was 22 years old, and the oldest was 86 years old. Out of the 60 patients, 38.33% (*n* = 23) died during follow-up. The mean age of the patients who died was 65.7 (SD = 12.98; minimum = 32; maximum = 86). On the other hand, the mean age of the patients who survived was 52.35 (SD = 15.08; range: 22–82). As both groups passed the Shapiro–Wilk normality test (W = 0.9737, *p* = 0.516 for alive patients and W = 0.9506, *p* = 0.3006 for deceased patients), the age is presented visually as mean with SD (Figure 1).

The age difference between the two groups was statistically significant (*p* = 0.0009, unpaired *t*-test).

Regarding the gender of the patients, 40% (*n* = 24) were male, and 60% (*n* = 36) were female. In the survivor group, 43.24% (*n* = 16) were male and 56.76% (*n* = 21) were female, resulting in a male-to-female ratio of 1:1.31. Regarding the patients who died during follow-up, 34.78% (*n* = 8) of the patients were male, and 65.22% (*n* = 15) were female, resulting in a male-to-female ratio of 1:1.88. This difference was not statistically significant (*p* = 0.5154; Chi-square test).

Additionally, the living environment of the patients was also noted. In this context, 31.67% (*n* = 19) of the patients resided in rural areas, while 68.33% (*n* = 41) in urban areas. In the deceased group, 43.58% (*n* = 10) of the patients came from rural areas, and 56.52% (*n* = 13) from urban areas. In patients who remained alive, 24.32% (*n* = 9) resided in a rural environment, and 75.68% (*n* = 41) in an urban environment. However, these differences were not statistically significant (*p* = 0.1573, Fisher’s exact test).

Concerning the location of the tumors, 23% (*n* = 14) were in the stomach, and the rest (*n* = 46) were in the small and large intestine, including 6 cases in the appendix (Table 2).

**Table 2:** Site-specific and gender frequency.

Site	Number of cases	Male-to-female ratio
Stomach	14	9:5
Small intestine	19	11:8
Ileocecal valve	1	0:1
Colo-rectal	20	3:17
Appendix	6	1:5

In patients who survived, 16.22% (*n* = 6) of the tumors were found in the stomach, while 83.78% (*n* = 31) were in the small and large intestines. In contrast, among patients who did not survive, 34.78% (*n* = 8) of the tumors were of gastric origin, and 65.22% (*n* = 15) were of intestinal origin. This difference was not statistically significant (*p* = 0.1234, Fisher’s exact test).

**Figure 2:**
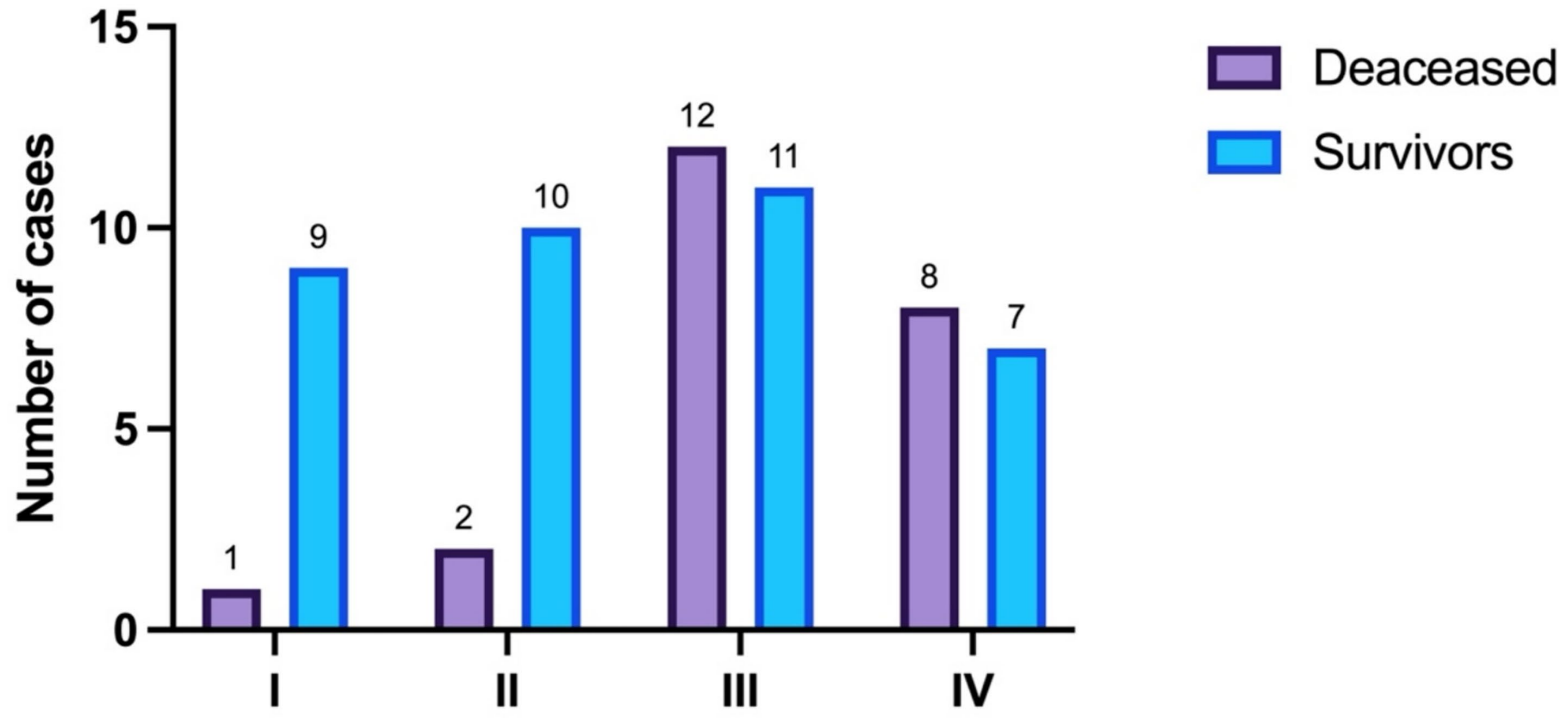
Disease stage distribution.

Regarding disease stage, its distribution is presented in Table 3 and visually in Figure 2. The difference in distribution is statistically significant (*p* = 0.034, Fisher’s exact test).

**Table 3:** Disease stage.

Stage	Total	Survivors	Deceased
I	16.67% (*n* = 10)	24.32% (*n* = 9)	4.35% (*n* = 1)
II	20% (*n* = 12)	27.03% (*n* = 10)	8.7% (*n* = 2)
III	38.33% (*n* = 23)	29.73% (*n* = 11)	52.17% (*n* = 12)
IV	25% (*n* = 15)	18.92% (*n* = 7)	34.78% (*n* = 8)

To proceed, the following histopathological parameters were evaluated: the diagnosis (NET vs. NEC), the presence of lympho-vascular invasion and the presence of perineural invasion (Table 4).

**Table 4:** The histopathological characteristics of the NENs.

Parameters	Survivors		Deceased		Total		*p* value (Fisher’s exact test)
Diagnosis	13.51% NEC (*n* = 5)	86.49% NET (*n* = 32)	52.17% NEC (*n* = 12)	47.83% NET (*n* = 11)	28.33% NEC (*n* = 17)	71.64% NET (*n* = 43)	**0.0025**
Lympho-vascular invasion	37.84% (*n* = 14)		60.87% (*n* = 14)		46.67% (*n* = 28)		0.1122
Perineural invasion	27.03% (*n* = 10)		52.17% (*n* = 12)		36.67% (*n* = 22)		0.0595

The values in bold indicate statistically significant results.

**Figure 3:**
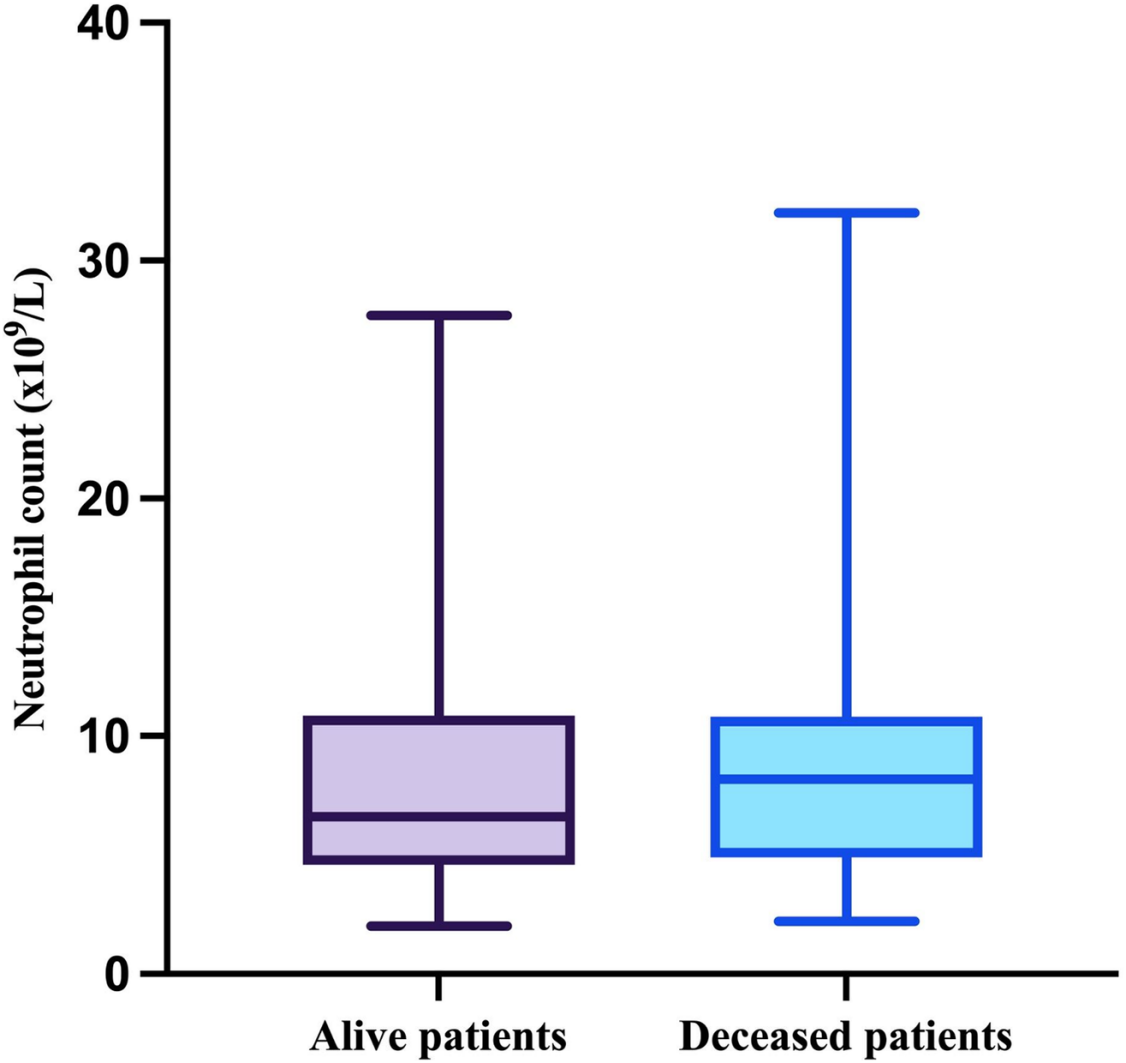
The median, minimum, and maximum values of the neutrophil count based on patient outcome.

The primary focus of this research was to assess the prognostic value of blood inflammatory markers. To begin with, the median value of the absolute neutrophil count in patients who survived was 6.6 × 10^9^/L (minimum = 2 × 10^9^/L, maximum = 27.7 × 10^9^/L), while the median value in the deceased group was 8.2 × 10^9^/L (minimum = 2.2 × 10^9^/L, maximum = 32 × 10^9^/L). As neither the neutrophil count in alive patients (W = 0.8213, *p* < 0.0001) nor in deceased patients (W = 0.7941, *p* = 0.0003) passed the Shapiro–Wilk normality test, they are presented as boxes with median and interquartile range; and whiskers indicating minimum and maximum values (Figure 3).

The difference in neutrophil counts between the two groups was not statistically significant (*p* = 0.4662, Mann–Whitney test).

**Figure 4:**
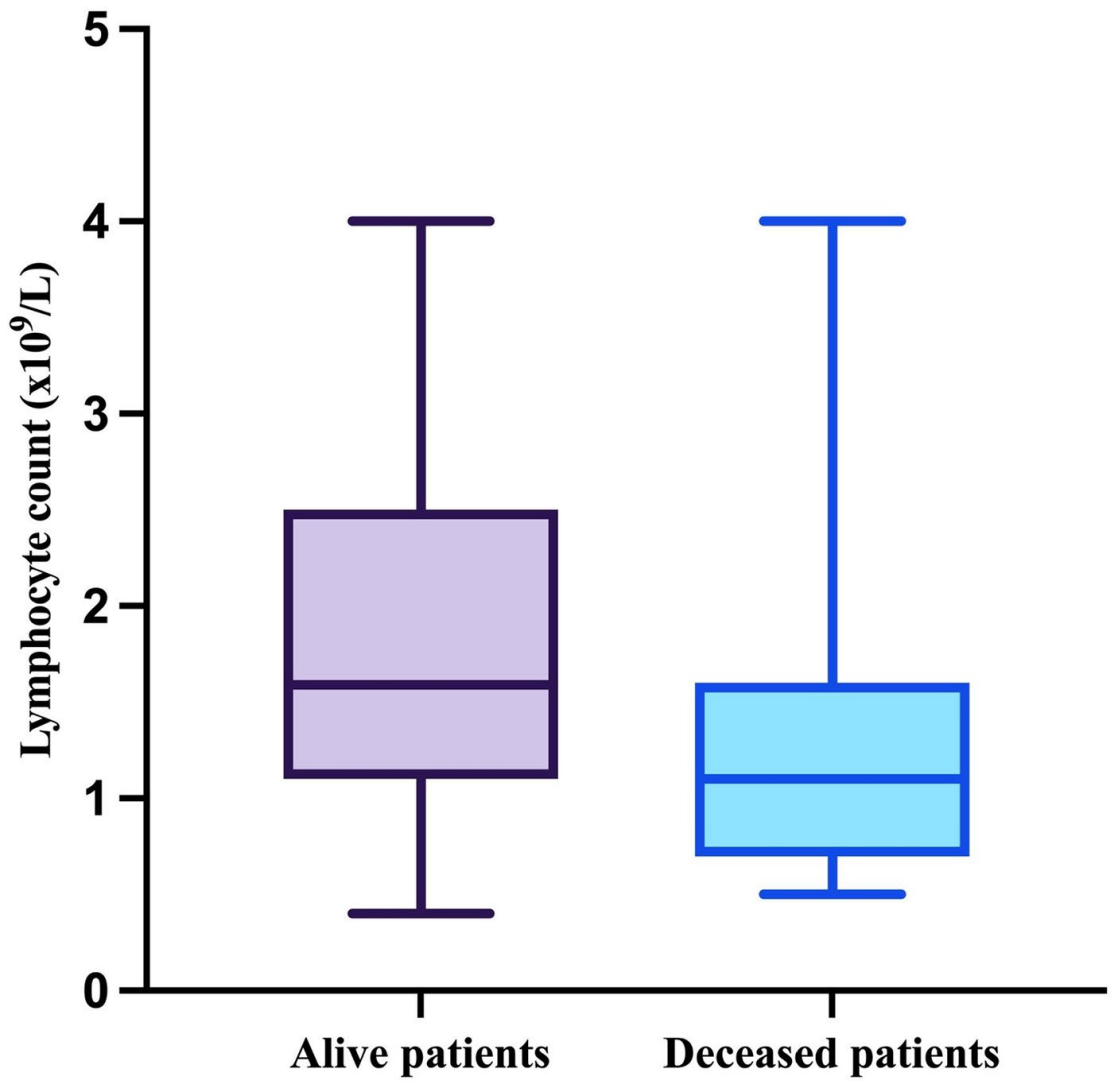
The median, minimum, and maximum values of the lymphocyte count based on patient outcome.

To go on, the median value of the lymphocyte counts in patients who survived was 1.59 × 10^9^/L (minimum = 0.4 × 10^9^/L, maximum = 4 × 10^9^/L), whereas in patients who died during follow-up, it was 1.1 × 10^9^/L (minimum = 0.5 × 10^9^/L, maximum = 4 × 10^9^/L) (Figure 4).

As the results of the Shapiro–Wilk test indicated that both lymphocyte count in alive patients (W = 0.9241, *p* = 0.0147) and in deceased patients (W = 0.7943, *p* = 0.0003) significantly deviated form normality, they were compared using the Mann–Whitney test, which demonstrated a significant difference between the two groups (*p* = 0.0092).

**Figure 5:**
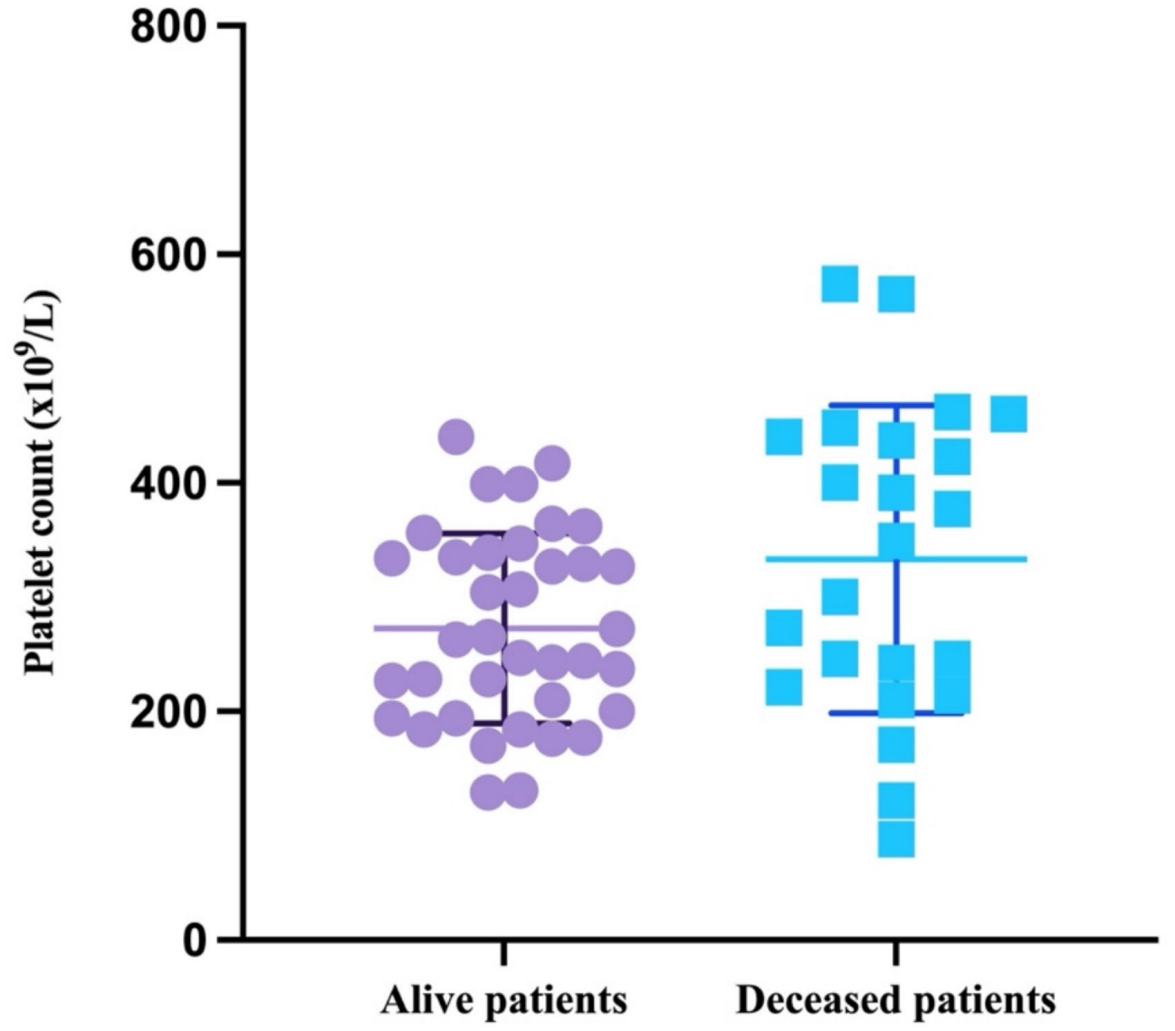
Scatter plot representing the mean with SD of the platelet count based on patient outcome.

Interestingly, the platelet counts in both alive (W = 0.9631, *p* = 0.252) and deceased patients (W = 0.9621, *p* = 0.5076) passed the Shapiro–Wilk normality test and are therefore as mean with SD. The mean value in the survivor group was 272.7×10^9^/L (SD = 83.29; minimum = 129; maximum = 444), and the mean value for the deceased patients was 333 (SD = 133.7; minimum = 88; maximum = 574) (Figure 5).

The difference in the platelet counts between the two groups was statistically significant (*p* = 0.0358, unpaired *t*-test).

**Figure 6:**
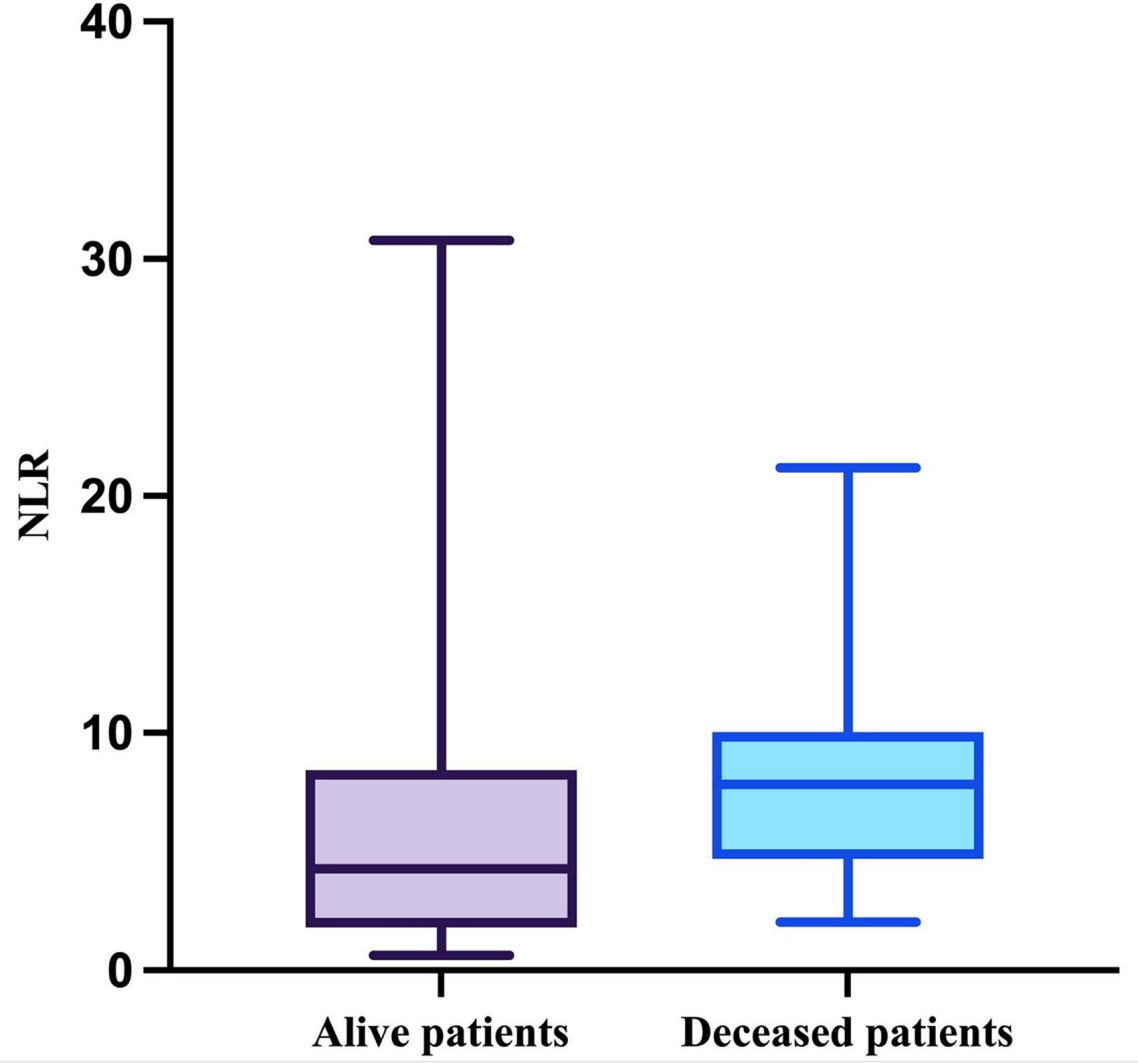
The median, minimum, and maximum values of NLR based on patient outcome.

Regarding the NLR, this parameter passed the Shapiro–Wilk normality test in the deceased group (W = 0.9164, *p* = 0.0557) but not in the survivor group (W = 0.7830, *p* < 0.0001). The median value of the NLR in patients who survived during follow-up was 4.286 (minimum = 0.6286; maximum = 30.78), while the median value in patients who died was 7.833 (minimum = 2.042; maximum = 21.2), with a mean of 7.977 (SD = 4.504) (Figure 6).

The difference in NLR between the two groups was statistically significant (*p* = 0.0355, Mann–Whitney test).

**Figure 7:**
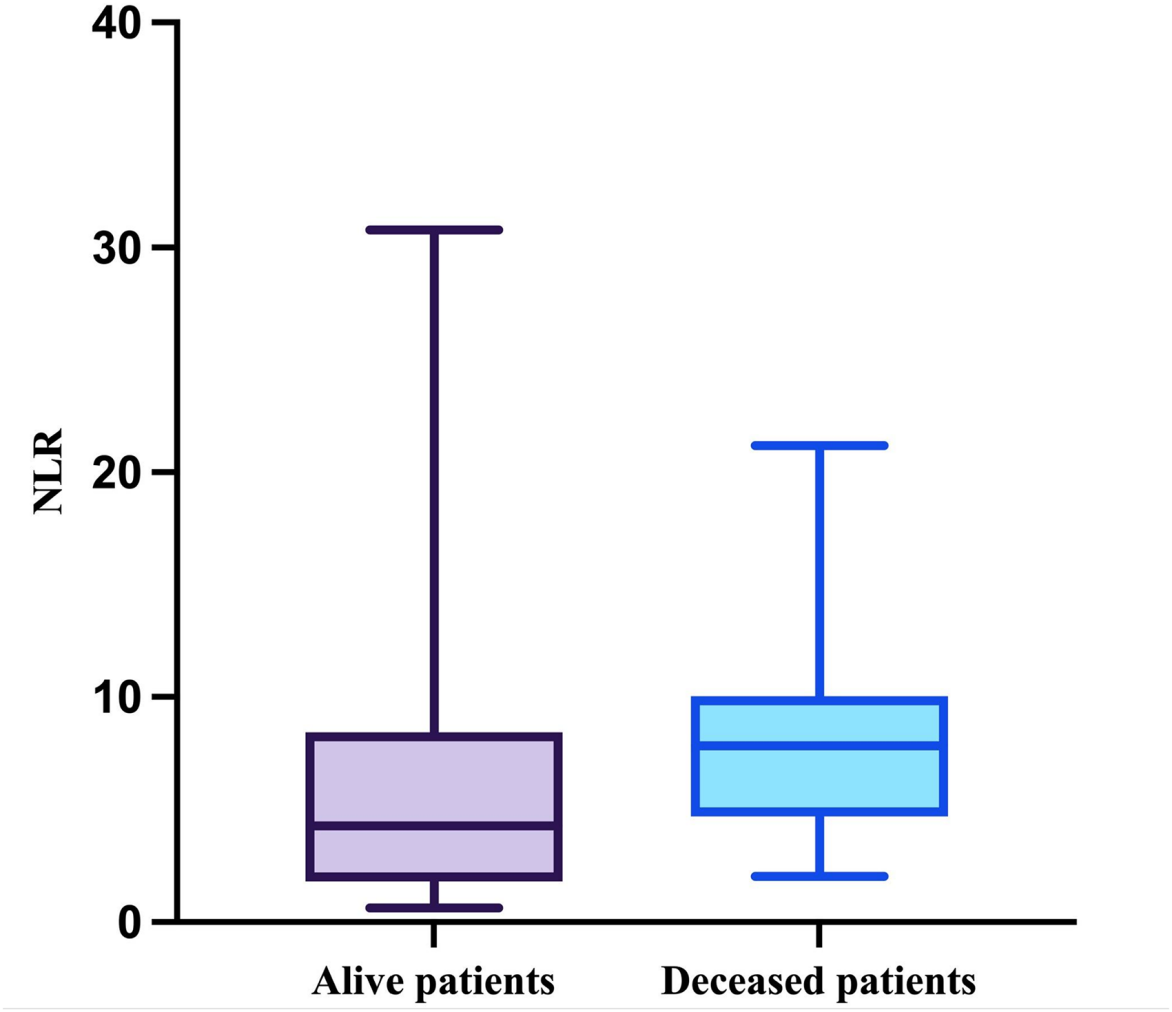
The median, minimum, and maximum values of PLR based on patient outcome.

Similarly, the PLR in the deceased group presented a normal distribution (W = 0.9804, *p* = 0.9804, Shapiro–Wilk test) while the PLR in the survivor group did not (W = 0.8157, *p* = 0.0083, Shapiro–Wilk test). Therefore, the median value in patients who survived was 164.5 (minimum = 33.95; maximum = 526) and 300 (minimum = 55; maximum = 640) in the deceased group, with a mean of 302.1 (SD = 134.2) (Figure 7). This difference was statistically significant (*p* = 0.0026, Mann–Whitney test).

**Figure 8:**
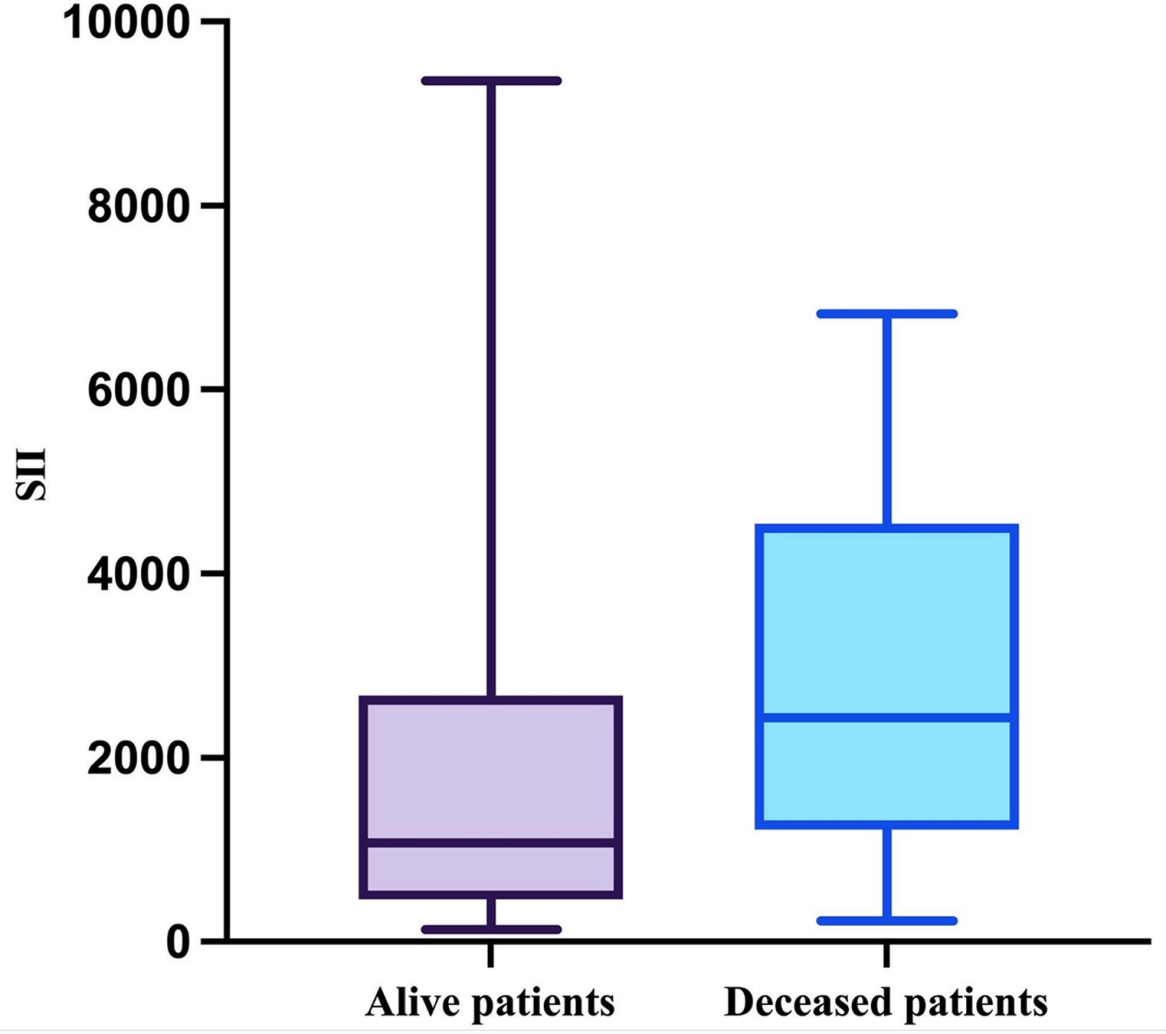
The median, minimum, and maximum values of SII based on patient outcome.

Lastly, the SII was evaluated. As expected, the SII in the deceased group passed the Shapiro–Wilk normality test (W = 0.9438, *p* = 0.2165) but the SII in the other group did not (W = 0.7636, *p* < 0.0001). The median value in the survivor group was 1,075 (minimum = 132; maximum = 9,356), and in the deceased group, it was 2,433 (minimum = 220; maximum = 6,820) with a mean of 2,813 (SD = 1868) (Figure 8). This difference was also statistically significant (*p* = 0.0161, Mann–Whitney test).

To assess the discriminatory performance of inflammatory markers, we performed Receiver Operating Characteristic (ROC) curve analysis. The optimal cut-off values were determined by the Youden Index. Using these cut-offs, patients were stratified into low and high groups (Table 5).

**Table 5:** Optimal cut-off values for the inflammatory markers.

Variable	Optimal Cut-off	AUC	Sensitivity	Specificity
Neutrophils	>8.20	557	52.2%	67.6%
Lymphocytes	≤1.30	699	73.9%	70.3%
Platelets	>377	647	47.8%	89.2%
NLR	>3.67	662	91.3%	43.2%
PLR	>235.3	729	73.9%	73.0%
SII	>1215.5	685	78.3%	56.8%

To go on, we performed univariate analysis for the inflammatory markers based on their cut-off values, together with the clinical, demographic and histopathologic parameters. Disease stage was analyzed by comparing advanced (III/IV) vs. Early (I/II) stages due to the reduced number of events in the early-stage category (Table 6).

**Table 6:** Univariate logistic regression.

Variable	OR	95% CI	*p* value
Age	1.07	1.03–1.13	**0.0006**
Gender (male)	0.7	0.23–2.03	0.5139
Environment (urban)	0.42	0.13–1.27	0.1235
Disease stage (III/IV)	7.04	1.78–27.81	**0.005**
Tumor location (intestinal)	0.36	0.1–1.224	0.1021
Diagnosis (NEC)	6.98	2.1–26.4	**0.0013**
Lymphovascular invasion (absent)	0.39	0.13–1.12	0.0812
Perineural invasion (present)	2.95	0.99–9.04	0.0501
Neutrophils (>8.2)	2.27	0.78–6.62	0.132
Lymphocytes (≤1.30)	6.70	2.08–21.52	**0.001**
Platelets (>377)	7.56	2.02–28.35	**0.003**
NLR (>3.67)	8.00	1.63–39.21	**0.01**
PLR (>253.3)	7.65	2.35–24.90	**<0.001**
SII (>1215.5)	4.72	1.44–15.46	**0.01**

Reference categories: female sex, rural environment, early stage (I/II), gastric tumor location, neuroendocrine tumor (NET), presence of lymphovascular invasion, absence of perineural invasion. The values in bold indicate statistically significant results.

As observed in Table 6, the age of the patients is a significant prognostic factor in patients with NENs, while the gender, the living environment and the location of the tumors have no significant predictive value in univariate analyses. Advanced TNM stage (III/IV) was significantly associated with increased mortality compared to early-stage disease (I/II) (OR: 7.04; 95% CI: 1.78–27.81; *p* = 0.005). As expected, the diagnosis of NEC versus NET is a significant negative prognostic factor, however the other two analyzed parameters (lymphovascular and perineural invasion) portend no significant prognostic value. Finally, lymphopenia (OR: 6.70; *p* = 0.001) and thrombocytosis (OR: 7.56; *p* = 0.003) were significant risk factors, whereas neutrophil count alone did not reach statistical significance (*p* = 0.132). High PLR (>235.3) was strongly associated with adverse outcomes (OR: 7.65; 95% CI: 2.35–24.90; *p* < 0.001). Elevated NLR (>3.67) was also a significant predictor of mortality (OR: 8.00; 95% CI: 1.63–39.21; *p* = 0.01). Patients with elevated SII (>1215.5) had a significantly increased risk of mortality (OR: 4.72; 95% CI: 1.44–15.46; *p* = 0.01).

Lastly, we performed a multivariate logistic regression analysis that included the demographic and clinical data, together with the diagnosis of NET vs. NEC, as well as SII. The other clinical, histopathological and inflammatory markers were excluded from this analysis to avoid collinearity. Due to the limited number of events in early-stage disease and to avoid model overfitting (maintaining a valid events-per-variable ratio), TNM stage was not included in the final multivariate logistic regression analysis (Table 7).

**Table 7:** The multivariate logistic regression analysis for death as a binary outcome.

Variable	OR	95% CI	*p* value
Age	1.06	1.01–1.12	**0.026**
Gender (male)	0.58	0.13–2.54	0.469
Tumor location (intestinal)	0.19	0.04–1.02	0.053
Diagnosis (NEC)	6.2	1.33–28.99	**0.02**
SII (>1215.5)	5.32	1.23–22.98	**0.025**

Reference categories: female sex, gastric tumor location, neuroendocrine tumor (NET). The values in bold indicate statistically significant results.

Based on the results of the multivariate analysis, the age of the patients, the diagnosis of NEC, and SII retained their strong negative prognostic value. Interestingly, the intestinal location of the tumors showed a borderline trend toward better prognosis, but the result was not statistically significant.

Finally, to evaluate the stability of the prognostic value of SII across different clinical presentations and address tumor heterogeneity, a stratified analysis was performed based on anatomical site and histological diagnosis. The Odds Ratios represent the risk of mortality for patients with High SII (>1215.5) compared to Low SII within each specific subgroup (Table 8).

**Table 8:** Stratified analysis of the prognostic value of SII by tumor location and histological subtype.

Subgroup	Total	Events (deaths)	SII OR	95%CI	*p* value
Tumor location					
Intestinal	46	15	5.54	1.30–23.67	**0.021**
Gastric	14	8	3.00	0.31–28.84	0.341
Histological subtype					
NET	43	11	5.79	1.07–31.16	**0.041**
NEC	17	12	4.50	0.49–41.25	0.183

The values in bold indicate statistically significant results.

When stratified by tumor location, elevated SII was a significant predictor of mortality in the intestinal subgroup (OR: 5.54, 95% CI: 1.30–23.67, *p* = 0.021). In the gastric subgroup (*n* = 14), while statistical significance was not reached due to the limited sample size (*p* = 0.341), a consistent positive association was observed (OR: 3.00), suggesting a similar risk profile.

Regarding histological subtype, in patients with NETs, high SII significantly predicted adverse outcomes (OR: 5.79, 95% CI: 1.07–31.16, *p* = 0.041). Similarly, in the NEC subgroup, the Odds Ratio indicated a strong risk association (OR: 4.50), although this did not reach statistical significance (*p* = 0.183), but this lack of significance should be interpreted in the view of reduced number of NEC cases (*n* = 17).

Collectively, these stratified data indicate that the prognostic signal of SII is robust within the major subgroups and maintains a consistent trend across the heterogeneous population.

## 4 Discussion

The role of inflammatory biomarkers is increasingly acknowledged as pivotal in various malignancies, including NENs. A bidirectional relationship exists between chronic inflammation and the pathogenesis of NEN. Prolonged inflammatory states can lead to excessive stimulation of neuroendocrine cells, potentially resulting in hyperplasia and subsequent neoplastic transformation. Moreover, the neoplasm itself can induce and sustain a chronic inflammatory response, further complicating the tumor microenvironment and influencing disease progression (8).

In recent years, an increasing amount of research has investigated the potential of hematological parameters as effective biomarkers for patients with NENs, aiming to elucidate the relationships between various blood count indices and the progression of the disease, as well as patients’ responses to treatment in this specific population.

In this retrospective study, we investigated the prognostic role of systemic inflammatory markers, specifically NLR, PLR, and SII, in conjunction with demographic and histopathological parameters, in a cohort of patients diagnosed with NENs. Our findings demonstrate that age (OR = 1.06, 95% CI 1.01–1.12, *p* < 0.026), diagnosis of NEC compared to NET (OR = 6.2, 95% CI 1.33–22.98, *p* < 0.025), and systemic inflammatory status assessed by SII with the cut-off value of 1215.5 (OR = 5.32, 95% CI 1.23–22.98, *p* < 0.025) are significant predictors of mortality after adjustment for selected clinicopathological variables.

Several recent studies corroborate our findings regarding the prognostic significance of systemic inflammatory markers in NENs. In a cohort of patients with nonfunctioning pancreatic neuroendocrine tumors (pNETs) undergoing surgical resection, higher SII was found to be an independent predictor of poorer overall survival, with a multivariate odds ratio (OR) of 8.43 (95% CI 3.19–22.72, *p* < 0.0001) (9). Similarly, in pulmonary neuroendocrine carcinomas, preoperative SII, NLR, and PLR were all significantly associated with survival, with SII demonstrating particularly robust prognostic value (10). In gastric neuroendocrine neoplasms (g-NENs), elevated PLR was also shown to have independent predictive value: patients in the highest PLR quartile (≥187) had approximately 1.8-fold greater risk of mortality compared to those in lower quartiles, and a standard-deviation increase in PLR was associated with a 54–67% increase in all-cause mortality in fully adjusted models (11).

The significant negative prognostic impact of NEC compared with NEN aligns with existing data. Poorly differentiated NECs are associated with more aggressive behavior, higher rates of cell proliferation, and limited responses to treatment. Although vascular or perineural invasion was more common in patients who died, it did not achieve statistical significance. This may be attributed to the small sample size and the variability of tumor sites included in our cohort.

Among the systemic inflammatory markers, lymphopenia and thrombocytosis were correlated with a worse prognosis. Lymphocytes play a pivotal role in mediating antitumor immune surveillance, and their depletion indicates a compromised immune response in the host (12). Platelets release factors that support tumor growth, angiogenesis, and play a significant role in tumor progression, acting as a shield for circulating tumor cells that allows them to evade immune detection. This mechanism not only enhances the survival of these cells in circulation but also accelerates metastatic spread, significantly contributing to the aggressiveness of malignancies (13). These mechanisms support our observation that both a low lymphocyte count ≤1.30 (OR = 6.7; 95% CI: 2.08–21.52; *p* = 0.001) and an elevated platelet count >377 (OR = 8.00; 95% CI: 2.02–21.52, *p* = 0.003) were significantly associated with increased mortality in patients with NENs.

In our univariate analysis of derived indices, we found that NLR > 3.67 and PLR > 253.3 were significantly higher in the deceased group, with an OR of 8.00 (95% CI: 1.63–39.21; *p* = 0.01) and 7.65 (95% CI: 2.35–24.9; *p* < 0.001), respectively. Additionally, SII > 1215.5 also demonstrated prognostic significance in univariate analysis, with an OR of 4.72 (95% CI: 1.44–15.46; *p* = 0.01). Our results reinforce the value of SII as a significant and cost-effective prognostic tool in the clinical management of NENs. Similar observations have been made in other malignancies, where SII has proven to be a strong prognostic marker, reflecting the combined effects of lymphopenia, neutrophilia, and thrombocytosis (14–16).

Another noteworthy finding is the trend of an association between intestinal NENs and a lower risk of death in comparison to gastric neoplasms in the multivariate analysis, even though this association did not reach statistical significance in our study (OR = 0.19; 95%CI: 0.04–1.02; *p* = 0.053). This observation is concordant with previous studies indicating that small intestinal and colorectal tend to have more favorable outcomes than those located in the gastric or pancreatic regions. These differences may reflect variations in biological behavior, stage at diagnosis, and response to treatment (17–19).

Corroborating all the results obtained, we consider that easily accessible, cost-effective, and non-invasive inflammatory markers, may serve as useful complements to established clinical and pathological prognostic factors in NENs. This approach could be beneficial in clinical settings where advanced molecular diagnostics are not readily accessible. However, we must note that our study has certain limitations, including its retrospective design, a relatively small sample size, and the absence of Ki-67 index or staging data in the multivariate analysis. While we adjusted for tumor location and age, the cohort size precluded a granular subgroup analysis based on WHO differentiation grades. Additionally, given the relatively small number of events, the multivariate analysis may be affected by overfitting and limited precision, as reflected by wide confidence intervals. Therefore, these findings should be considered exploratory and require confirmation in larger, prospective studies. Lastly, time-to-event data were not available for all patients; therefore, survival analyses using Cox regression could not be performed, and mortality was analyzed as a binary outcome. Consequently, it is essential to conduct future prospective multicenter studies to corroborate these findings and to incorporate inflammatory biomarkers into risk stratification frameworks for NENs.

## 5 Conclusion

This study shows that age, a diagnosis of NEC, and an elevated SII are significant negative prognostic factors in patients with neuroendocrine neoplasms. In contrast, intestinal tumor location is associated with lower mortality rates. Among the systemic inflammatory markers analyzed, the SII and PLR demonstrated superior prognostic value compared to NLR, highlighting the contribution of platelet counts in reflecting tumor–host interactions. Given their accessibility, simplicity, and low cost, these indices may serve as valuable adjuncts to traditional clinicopathological parameters in the risk stratification and management of NEN patients. Validation through prospective, multicenter studies is warranted to establish their role in clinical practice and to integrate them into standardized prognostic models.

## Data Availability

The raw data supporting the conclusions of this article will be made available by the authors, without undue reservation.
